# Supplementary figures and images for: Transcriptome analysis revealed misregulated gene expression in blastoderms of interspecific chicken and Japanese quail F1 hybrids
Source: PLoS One. 2020 Oct 12;15(10):e0240183. doi: 10.1371/journal.pone.0240183 (PMC7549780; doi:10.1371/journal.pone.0240183)

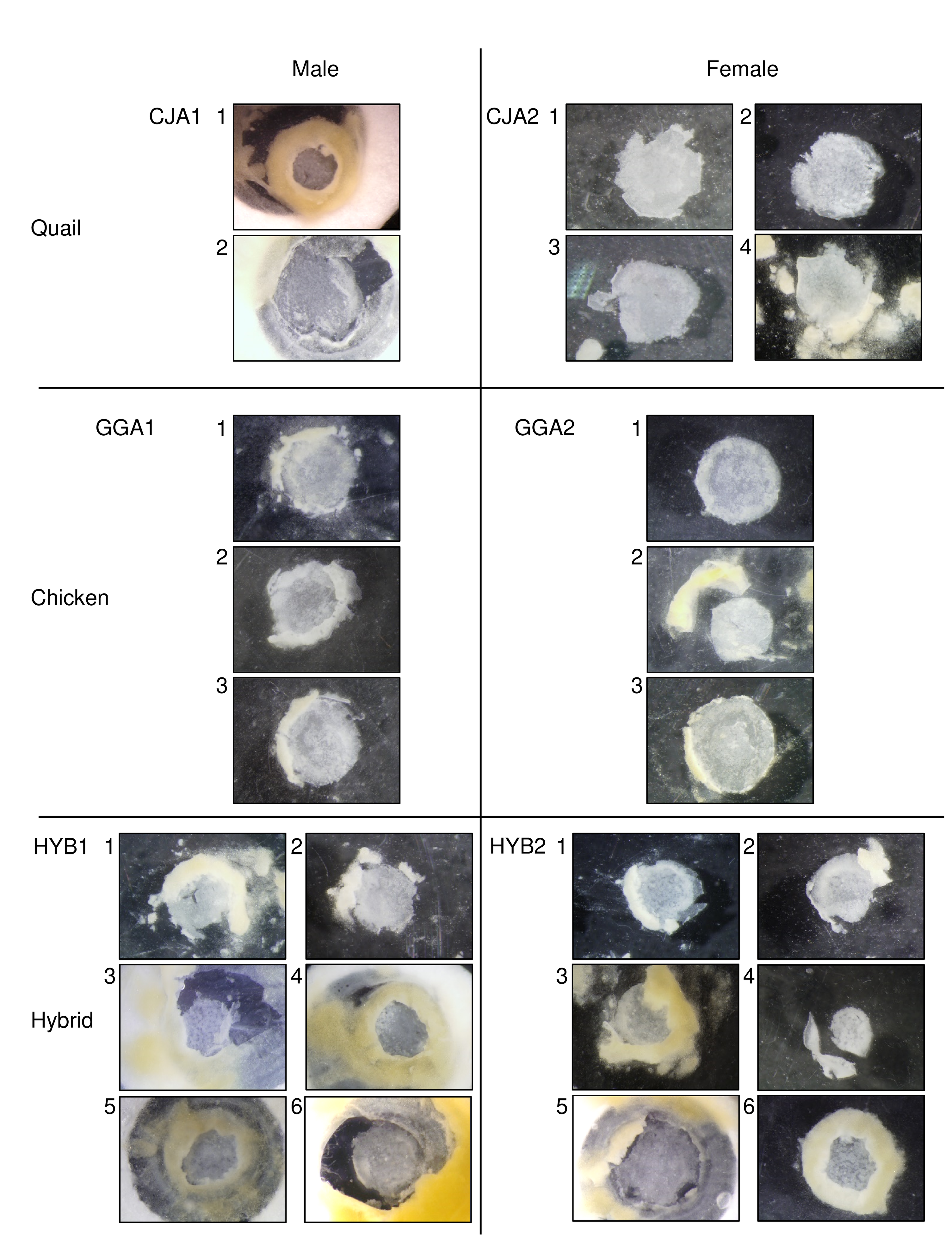

Supplement: S1 Fig — Images of blastoderms are shown with their sample numbers. (TIF) [file pone.0240183.s004.tif]

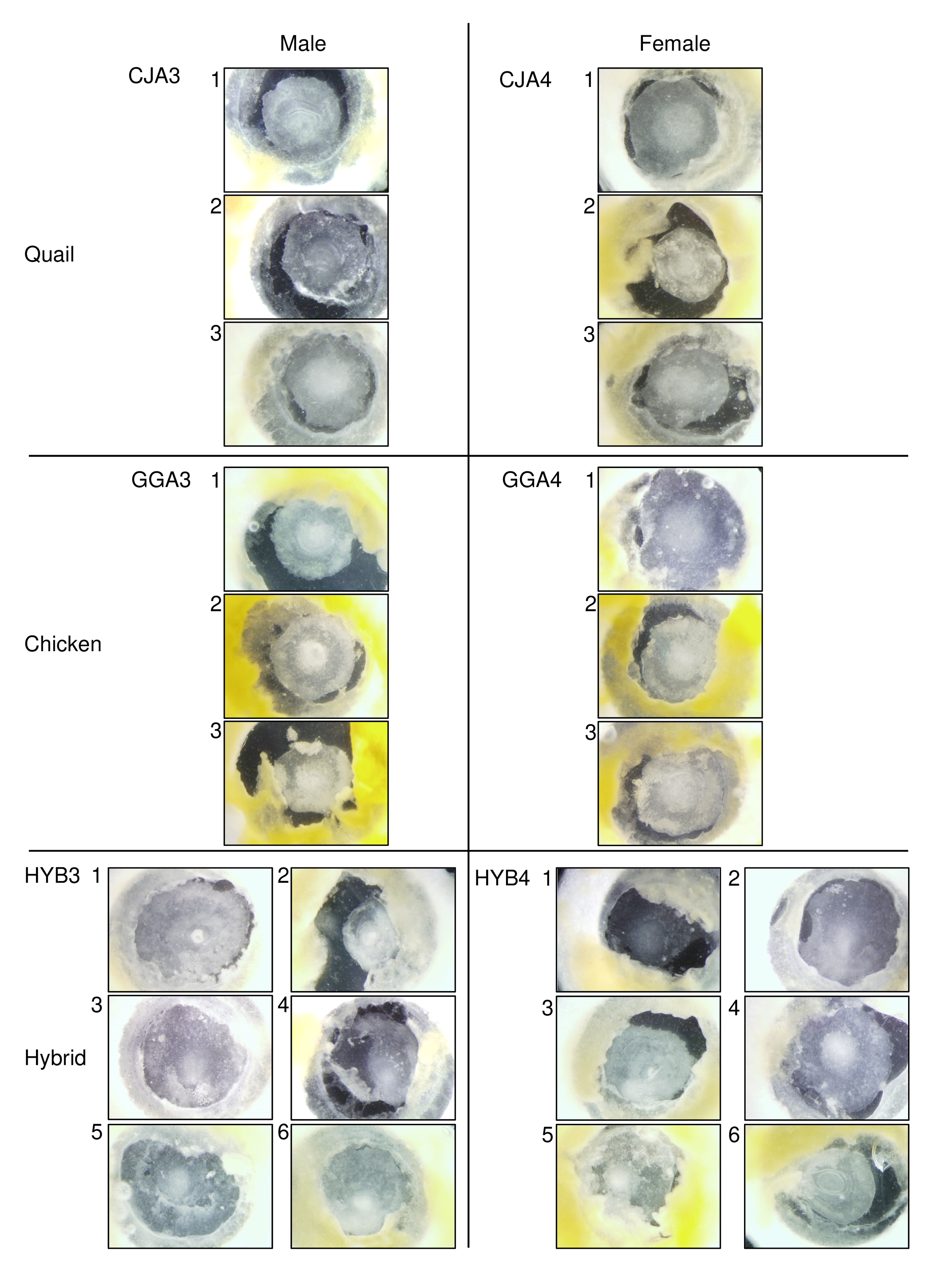

Supplement: S2 Fig — Images of blastoderms are shown with their sample numbers. (TIF) [file pone.0240183.s005.tif]

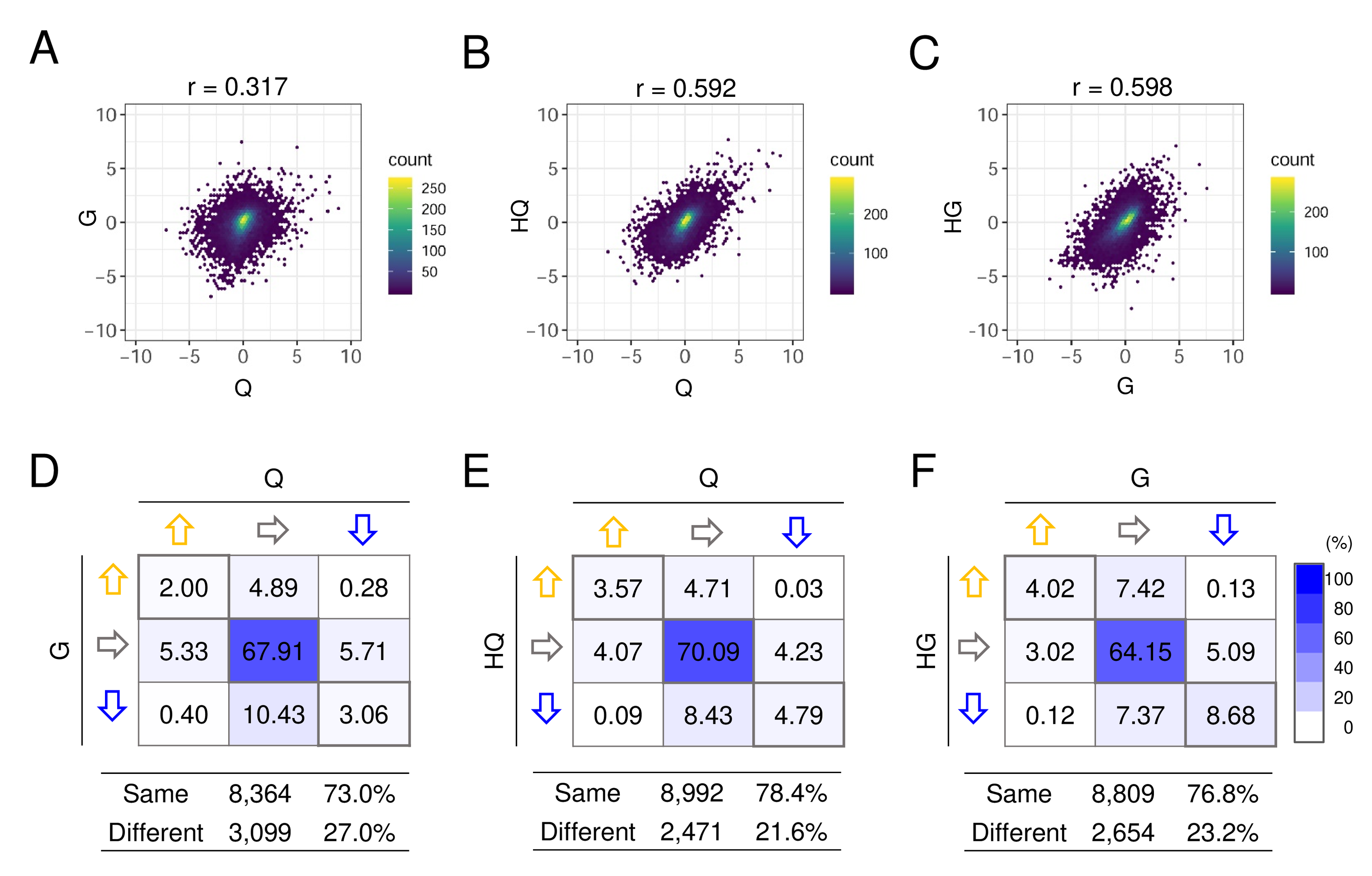

Supplement: S3 Fig — A–C. Comparison of gene expression changes [log2(fold change)] between quail (‘Q’) and chickens (‘G’) (A), between quail (‘Q’) and quail-derived alleles in the hybrids (‘HQ’) (B), and between chickens (“G”) and chicken-derived alleles in the hybrids (‘HG’) (C). Pearson’s correlation efficient (r) is indicated above the graphs. D–F. Comparison of the direction of gene expression changes between quail and chickens (D), between quail and quail-derived alleles in the hybrids (E), and between chickens and chicken-derived alleles in the hybrids (F). The number in each rectangle indicates the percentage of genes. Percentages of genes that exhibited the same direction of expression changes are indicated in bold-lined rectangles. Numbers and percentages of genes exhibiting the same or different directions of expression change are shown in the tables. Color scale at the far right shows the percentage of genes. (TIF) [file pone.0240183.s006.tif]

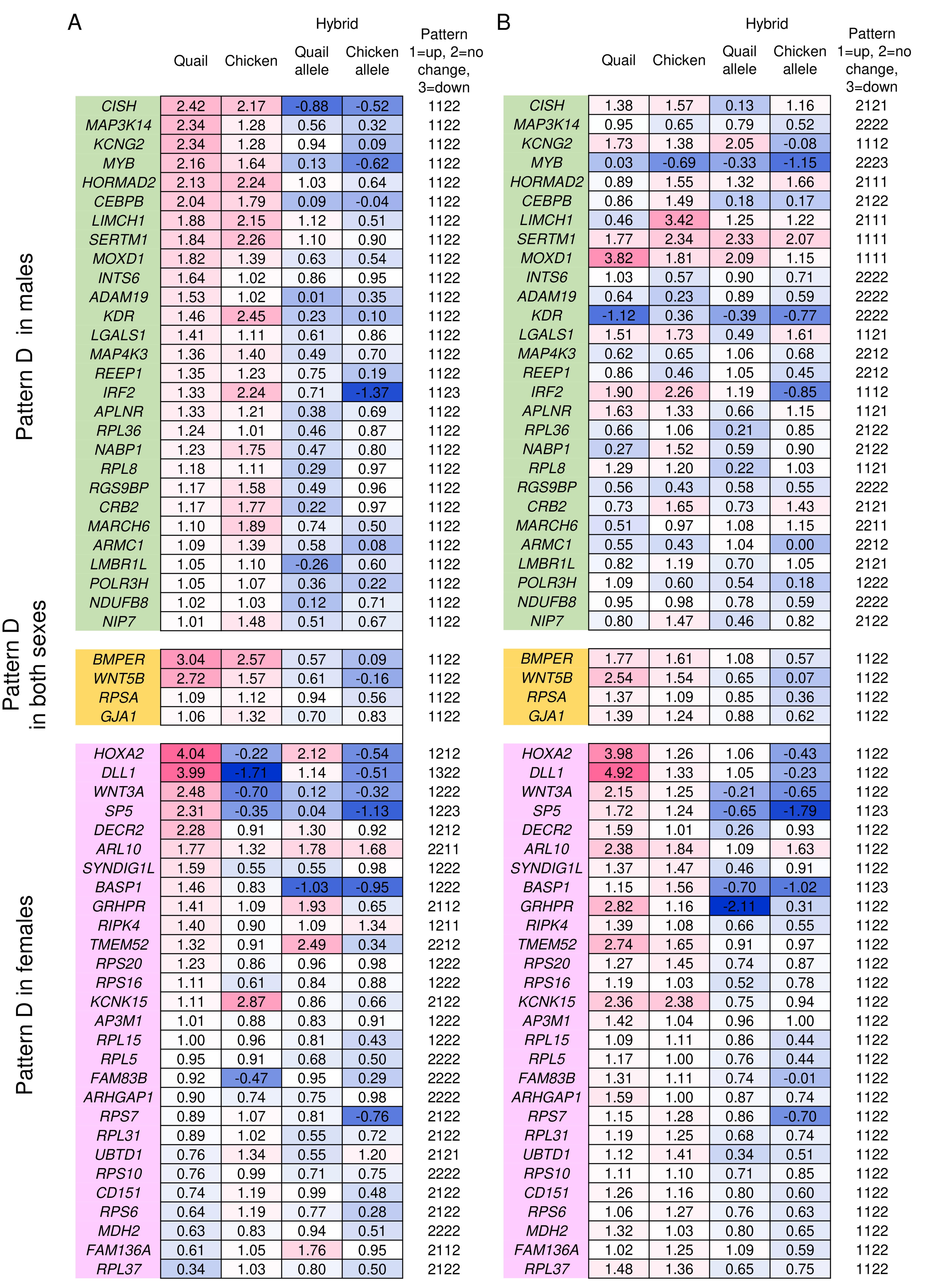

Supplement: S4 Fig — A, B. Patterns of expression changes of the 60 genes in male (A) and female (B) embryos. (TIF) [file pone.0240183.s007.tif]

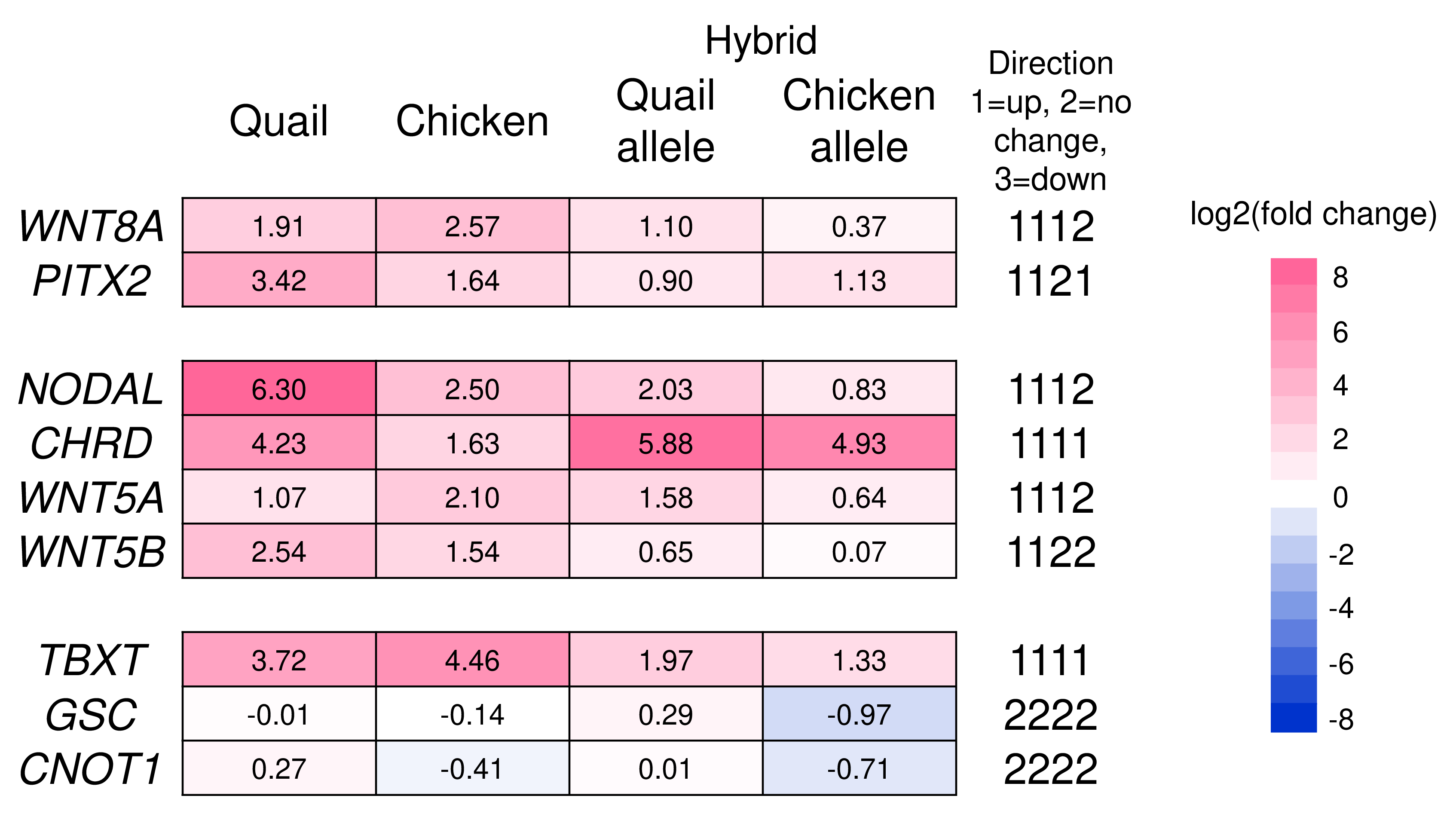

Supplement: S5 Fig — (TIF) [file pone.0240183.s008.tif]
