# Supplementary material for: Transcriptome analysis revealed misregulated gene expression in blastoderms of interspecific chicken and Japanese quail F1 hybrids
Source: PLoS One. 2020 Oct 12;15(10):e0240183. doi: 10.1371/journal.pone.0240183 (PMC7549780; doi:10.1371/journal.pone.0240183)
Supplement: S1 Table. The number of cells with abnormal number of chromosomes — (PDF) [file pone.0240183.s009.pdf]

S1 Table Number of cells with abnormal number of chromosomes.

ID	Sex	Chromosome number	Number of observed cells	Abnormal
10-4-26 (3 day embryo)	male	1	41	0
		2	41	0
		3	26	0
		4	33	0
		5	34	0
		6	33	0
		7	25	0
		8	25	0
		9	34	0
		z	26	0
4-4-24 (3 day embryo)	female	1	41	0
		2	41	0
		3	29	0
		4	33	0
		5	37	0
		6	33	0
		7	37	0
		8	37	0
		9	37	0
		z	48	0
		w	48	0
2-10-18 (7 day embryo)	male	1	44	0
		2	26	0
		3	44	0
		4	53	0
		5	26	0
		6	53	0
		7	28	0
		8	28	0
		9	23	0
		z	23	0
4-10-18 (7 day embryo)	female	1	32	0
		2	44	0
		3	32	0
		4	29	0
		5	44	0
		6	29	0
		7	32	0
		8	32	0
		9	31	0
		z	48	0
		w	48	0
