# Supplementary material for: Transcriptome analysis revealed misregulated gene expression in blastoderms of interspecific chicken and Japanese quail F1 hybrids
Source: PLoS One. 2020 Oct 12;15(10):e0240183. doi: 10.1371/journal.pone.0240183 (PMC7549780; doi:10.1371/journal.pone.0240183)
Supplement: S2 Table — (PDF) [file pone.0240183.s010.pdf]

S2 Table Summary of patterns of gene expression changes.

Pattern of gene expression changes[a]		Male				Common[b]	
		Number of genes	Percentage	Number of genes	Percentage	Number of genes	Percentage[c]
All		11,575	100.0%	11,463	100.0%	6,613	58.3%
Q = G	11??, 22??, 33??	8,376	72.4%	8,364	73.0%	6,887	60.7%
Q ≠ G	12??, 13??, 21??, 23??, 31??, 32??	3,199	27.6%	3,099	27.0%	1,694	14.9%
Q = G, Q = HQ, G = HG	1111, 2222, 3333	6,285	54.3%	6,236	54.4%	4,817	42.5%
Q ≠ G, Q = HQ, G = HG	1212, 1313, 2121, 2323, 3131, 3232	718	6.2%	794	6.9%	284	2.5%
Upregulated in both Q and G, and in either HQ or HG	1112, 1113, 1121, 1131	41	0.4%	83	0.7%	9	0.1%
Upregulated in both Q and G, and in neither HQ nor HG	1122, 1123, 1132, 1133	32	0.3%	32	0.3%	4	0.03%
Unchanged in both Q and G, and in either HQ or HG	2221, 2223, 2212, 2232	1,650	14.3%	1,640	14.3%	627	5.5%
Unchanged in both Q and G, and in neither HQ or HG	2211, 2213, 2231, 2233	312	2.7%	266	2.3%	64	0.6%
Downregulated in both Q and G, and in either HQ or HG	3331, 3332, 3313, 3323	38	0.3%	70	0.6%	11	0.1%
Downregulated in both Q and G, and in neither HQ nor HG	3311, 3312, 3321, 3322	18	0.2%	37	0.3%	2	0.02%

[a] Expression changes of quail genes in quail, chicken genes in chickens, and quail- and chicken-derived alleles in hybrids. 1, upregulation; 2, no change; 3, downregulation.

[b] Genes that showed the same pattern of expression changes between males and females.

[c] Percentage of a total of 11,341 genes that were expressed in both sexes.
