# Supplementary material for: Transcriptome analysis revealed misregulated gene expression in blastoderms of interspecific chicken and Japanese quail F1 hybrids
Source: PLoS One. 2020 Oct 12;15(10):e0240183. doi: 10.1371/journal.pone.0240183 (PMC7549780; doi:10.1371/journal.pone.0240183)
Supplement: S3 Table — (PDF) [file pone.0240183.s011.pdf]

S3 Table Overrepresented GO-BP terms in 285 genes that were upregulated in male and/or female embryos of parental species.

GO biological process	Refference (*Gallus gallus*)	Input	Expected	Fold Enrichment	+/-	Raw P value	FDR
cytoplasmic translation	61	8	0.78	10.22	+	2.52E-06	2.67E-03
translation	290	21	3.72	5.64	+	4.92E-10	6.77E-06
peptide biosynthetic process	302	21	3.87	5.42	+	9.78E-10	4.49E-06
peptide metabolic process	389	22	4.99	4.41	+	1.39E-08	3.19E-05
cellular amide metabolic process	568	27	7.29	3.7	+	9.93E-09	2.73E-05
cellular process	12300	187	157.82	1.18	+	1.74E-05	1.41E-02
amide biosynthetic process	388	24	4.98	4.82	+	5.46E-10	3.76E-06
cellular nitrogen compound biosynthetic process	982	34	12.6	2.7	+	2.03E-07	2.54E-04
cellular biosynthetic process	1727	49	22.16	2.21	+	1.39E-07	2.13E-04
biosynthetic process	1843	52	23.65	2.2	+	8.45E-08	1.45E-04
organonitrogen compound biosynthetic process	976	38	12.52	3.03	+	1.64E-09	5.63E-06
organic substance biosynthetic process	1790	51	22.97	2.22	+	6.24E-08	1.23E-04
ribosomal small subunit biogenesis	65	7	0.83	8.39	+	3.50E-05	2.19E-02
carboxylic acid transmembrane transport	93	8	1.19	6.7	+	4.31E-05	2.58E-02
anion transport	443	18	5.68	3.17	+	2.51E-05	1.73E-02
ion transport	1023	35	13.13	2.67	+	1.73E-07	2.38E-04
ion transmembrane transport	724	24	9.29	2.58	+	2.90E-05	1.90E-02
transmembrane transport	1002	33	12.86	2.57	+	9.25E-07	1.06E-03
organic acid transmembrane transport	93	8	1.19	6.7	+	4.31E-05	2.47E-02
organic acid transport	207	12	2.66	4.52	+	2.39E-05	1.73E-02
carboxylic acid transport	206	12	2.64	4.54	+	2.28E-05	1.75E-02
organic anion transport	337	17	4.32	3.93	+	2.90E-06	2.49E-03
positive regulation of cell-cell adhesion	132	9	1.69	5.31	+	7.89E-05	4.35E-02
