# Supplementary material for: Transcriptome analysis revealed misregulated gene expression in blastoderms of interspecific chicken and Japanese quail F1 hybrids
Source: PLoS One. 2020 Oct 12;15(10):e0240183. doi: 10.1371/journal.pone.0240183 (PMC7549780; doi:10.1371/journal.pone.0240183)
Supplement: S4 Table — (PDF) [file pone.0240183.s012.pdf]

S4 Table GO-BP terms related to primitive streak formation and chromosome segregation.

GO terms
GO-BP terms including primitive streak formation-related GO-BP terms
[Inferred part_of relation] GO:0048856 anatomical structure development
[Inferred part_of relation] GO:0008150 biological_process
[Inferred part_of relation] GO:0007275 multicellular organism development
[Inferred part_of relation] GO:0032501 multicellular organismal process
[Inferred part_of relation] GO:0009653 anatomical structure morphogenesis
[Inferred part_of relation] GO:0032502 developmental process
[Inferred part_of relation] GO:0009790 embryo development
[Inferred part_of relation] GO:0007389 pattern specification process
[Inferred part_of relation] GO:0048598 embryonic morphogenesis
[Inferred part_of relation] GO:0003002 regionalization
[Inferred part_of relation] GO:0009952 anterior/posterior pattern specification
[Inferred part_of relation] GO:0009798 axis specification
[Inferred part_of relation] GO:0007369 gastrulation
[is_a relation] GO:0048646 anatomical structure formation involved in morphogenesis
[part_of relation] GO:0009948 anterior/posterior axis specification
[part_of relation] GO:0001702 gastrulation with mouth forming second
**[Current term] GO:0090009 primitive streak formation**
[part_of relation] GO:0090010 transforming growth factor beta receptor signaling pathway involved in primitive streak formation
[part_of relation] GO:0090011 Wnt signaling pathway involved in primitive streak formation
GO-BP terms including chromosome segregation-related GO-BP terms
[Inferred is_a relation] GO:0008150 biological_process
[is_a relation] GO:0009987 cellular process
**[Current term] GO:0007059 chromosome segregation**
[part_of relation] GO:0051304 chromosome separation
[negatively_regulates relation] GO:0051985 negative regulation of chromosome segregation
[is_a relation] GO:0098813 nuclear chromosome segregation
[positively_regulates relation] GO:0051984 positive regulation of chromosome segregation
[regulates relation] GO:0051983 regulation of chromosome segregation

GO-BP terms indicated in gray background were considered as those related to primitive streak formation and chromosome segregation in this study.
