# Supplementary material for: Transcriptome analysis revealed misregulated gene expression in blastoderms of interspecific chicken and Japanese quail F1 hybrids
Source: PLoS One. 2020 Oct 12;15(10):e0240183. doi: 10.1371/journal.pone.0240183 (PMC7549780; doi:10.1371/journal.pone.0240183)
Supplement: S5 Table — (PDF) [file pone.0240183.s013.pdf]

S5 Table Result of GO term enrichment analysis of 285 genes that were upregulated in male and/or female embryos of parental species (primitive streak formation- and chromosome segregation-related GO-BP terms).

GO biological process	Refference (*Gallus gallus*)	Input	Expected	Fold Enrichment	+/-	Raw P value	FDR	Genes
<u>Primitive streak formation-related</u>								
anterior/posterior pattern specification (GO:0009952)	190	7	2.44	2.87	+	1.29E-02	7.85E-01	*CDX1, DLL1, WNT3A, MSX2, CRB2, T, WNT5A*
axis specification (GO:0009798)	62	4	0.8	5.03	+	9.80E-03	7.03E-01	*CDX1, DLL1, PITX2, WNT5A*
gastrulation (GO:0007369)	118	6	1.51	3.96	+	5.00E-03	5.89E-01	*RPS6, WNT3A, CRB2, T, WNT5A, MMP9*
anatomical structure formation involved in morphogenesis (GO:0048646)	617	16	7.92	2.02	+	9.49E-03	7.26E-01	*PMP22, RECK, SLC40A1, DLL1, BMPER, PITX2, WNT3A, MSX2, CRB2, T, WNT5A, MMP9, ITGAV, S1PR1, RPS7, STRA6*
anterior/posterior axis specification (GO:0009948)	37	2	0.47	4.21	+	8.73E-02	1.00E+00	*CDX1, WNT5A*
gastrulation with mouth forming second (GO:0001702)	20	2	0.26	7.79	+	3.12E-02	9.34E-01	*CRB2, WNT5A*
primitive streak formation (GO:0090009)	5	1	0.06	15.59	+	7.37E-02	1.00E+00	*WNT5A*
<u>Chromosome segregation-related</u>								
chromosome segregation (GO:0007059)	227	2	2.91	0.69	-	1.00E+00	1.00E+00	*HORMAD2, PHB2*
nuclear chromosome segregation (GO:0098813)	188	2	2.41	0.83	-	1.00E+00	1.00E+00	*HORMAD2, PHB2*
