# Supplementary material for: Transcriptome analysis revealed misregulated gene expression in blastoderms of interspecific chicken and Japanese quail F1 hybrids
Source: PLoS One. 2020 Oct 12;15(10):e0240183. doi: 10.1371/journal.pone.0240183 (PMC7549780; doi:10.1371/journal.pone.0240183)
Supplement: S6 Table — (PDF) [file pone.0240183.s014.pdf]

S6 Table Summary of GO term enrichment analysis of 60 genes showing pattern D.

GO-BP term	Gene	Count	Fold Enrichment	P value	FDR
Overrepresented					
peptide biosynthetic process (GO:0043043)	*RPS6,RPL36,RPL8,RPS20,RPL37,RPL15,RPS16,RPSA,RPL5,RPS7*	10	11.16	2.3E-08	0.0002
translation (GO:0006412)	*RPS6,RPL36,RPL8,RPS20,RPL37,RPL15,RPS16,RPSA,RPL5,RPS7*	10	11.59	1.6E-08	0.0002
peptide metabolic process (GO:0006518)	*RPS6,RPL36,RPL8,RPS20,RPL37,RPL15,RPS16,RPSA,RPL5,RPS7*	10	8.61	2.4E-07	0.0008
amide biosynthetic process (GO:0043604)	*RPS6,RPL36,RPL8,RPS20,RPL37,RPL15,RPS16,RPSA,RPL5,RPS7*	10	8.68	2.2E-07	0.0010
ribosomal small subunit biogenesis (GO:0042274)	*RPS6,RPS10,RPS16,RPSA,RPS7*	5	25.31	2.2E-06	0.0051
positive regulation of sprouting angiogenesis (GO:1903672)	*KDR,DLL1,BMPER,APLNR*	4	50.63	1.9E-06	0.0054
cellular amide metabolic process (GO:0043603)	*RPS6,RPL36,RPL8,RPS20,RPL37,RPL15,RPS16,RPSA,RPL5,RPS7*	10	5.94	6.3E-06	0.0110
cellular nitrogen compound biosynthetic process (GO:0044271)	*RPS6,RPL36,RPL8,RPS20,RPL37,RPL15,RPS16,RPSA,RPL5,RPS7,POLR3H,MOXD1*	12	4.39	5.7E-06	0.0113
cell population proliferation (GO:0008283)	*RPS6,CEBPB,BMPER,IRF2,LMBR1L,WNT3A,CD151,FAM83B*	8	7.84	8.5E-06	0.0131
ribosome biogenesis (GO:0042254)	*RPS6,RPS10,RPS16,RPSA,RPL5,NIP7,RPS7*	7	9.48	9.9E-06	0.0138
regulation of sprouting angiogenesis (GO:1903670)	*KDR,DLL1,BMPER,APLNR*	4	28.62	1.6E-05	0.0197
ribosome assembly (GO:0042255)	*RPS10,RPSA,RPL5,NIP7*	4	24.84	2.6E-05	0.0305
organonitrogen compound biosynthetic process (GO:1901566)	*RPS6,RPL36,RPL8,RPS20,RPL37,RPL15,RPS16,RPSA,RPL5,RPS7,MOXD1*	11	4.03	3.2E-05	0.0345
cytoplasmic translation (GO:0002181)	*RPL36,RPL8,RPL15,RPSA*	4	21.58	4.4E-05	0.0440
Primitive streak formation-related					
gastrulation (GO:0007369)	*RPS6, WNT3A, CRB2*	3	8.37	5.9E-03	0.8910
anterior/posterior pattern specification (GO:0009952)	*DLL1, WNT3A, CRB2*	3	5.2	2.1E-02	1.0000
anatomical structure formation involved in morphogenesis (GO:0048646)	*DLL1, BMPER, WNT3A, CRB2, RPS7*	5	2.67	4.0E-02	1.0000
gastrulation with mouth forming second (GO:0001702)	*CRB2*	1	16.45	6.2E-02	1.0000
axis specification (GO:0009798)	*DLL1*	1	5.31	1.7E-01	1.0000
Chromosome segregation-related					
nuclear chromosome segregation (GO:0098813)	*HORMAD2*	1	0.438	1.8E+00	1.0000
chromosome segregation (GO:0007059)	*HORMAD2*	1	0.502	1.5E+00	1.0000

Overrepresented GO-BP terms and GO-BP terms related to primitive streak formation and chromosome segregation are shown. Processes that involve many ribosomal protein genes are indicated in gray.
