# Supplementary material for: Transcriptome analysis revealed misregulated gene expression in blastoderms of interspecific chicken and Japanese quail F1 hybrids
Source: PLoS One. 2020 Oct 12;15(10):e0240183. doi: 10.1371/journal.pone.0240183 (PMC7549780; doi:10.1371/journal.pone.0240183)
Supplement: Attachment — Submitted filename: Comments.docx [file pone.0240183.s015.docx]

The manuscript is about identifying the mis-regulated gene expression in blastoderms of interspecific chicken-Japanese quail F1 hybrids. Using transcriptome analysis, they have found that numerical abnormalities due to a segregation failure does not lead to the lethality of chicken–quail hybrid embryos, and that the developmental arrest at the pre-primitive streak stage in the hybrids is mainly caused by the downregulated expression of the genes involved in various biological processes such as translation and gastrulation.

The manuscript lacks the idiographic information in several parts and needs extensive restructuring.

Below are my specific comments mainly on the arrangement of materials and methods.

1. The manuscript is studying chicken (Male) and Japanese quail (Female) F1 hybrids. As well known, the fertilization and lethal rate of hybrids from the different combination is discrepancy. Have you looked at the gene expression of chicken (Female) and Japanese quail (Male) hybrids? If so, how about the result? If not, why?

2. Figures included in this manuscript are too blurry to look at the details. Please reupload figures with high resolution, especially Figure 1 and Figure 5.

3.The Figure legends in this manuscript should be corrected.

4. The statistics analysis needs to be corrected. Why were two different statistic methods used in the same analysis? For example, in Figure 2: the Tukey-Kramer test was used in panel A and B, while the Dunnet’s test was used in panel C and D. Besides, please explain why not use the Pearson’s test but Spearman’s rank in correlation coefficient analysis in Figure 4.

5. In Figure 2A, how do you explain the outliers in the Hybird group in panel A? In addition, the “A” is smaller than other alphabets, please correct the format of figure.

6. Discussion needs to be restructured explaining more about the impact of current findings in order to make the paper compelling for readers.
